# Geometric Convolutional Neural Network for Analyzing Surface-Based Neuroimaging Data

**DOI:** 10.3389/fninf.2018.00042

**Published:** 2018-07-06

**Authors:** Si-Baek Seong, Chongwon Pae, Hae-Jeong Park

**Affiliations:** ^1^Brain Korea 21 PLUS Project for Medical Science, College of Medicine, Yonsei University, Seoul, South Korea; ^2^Department of Nuclear Medicine, Radiology, and Psychiatry, Severance Hospital, College of Medicine, Yonsei University, Seoul, South Korea; ^3^Department of Cognitive Science, Yonsei University, Seoul, South Korea; ^4^Center for Systems and Translational Brain Sciences, Institute of Human Complexity and Systems Science, Yonsei University, Seoul, South Korea

**Keywords:** cortical thickness, surface-based analysis, geometric convolutional neural network, sex differences, Machine learning, neuroimage

## Abstract

In machine learning, one of the most popular deep learning methods is the convolutional neural network (CNN), which utilizes shared local filters and hierarchical information processing analogous to the brain’s visual system. Despite its popularity in recognizing two-dimensional (2D) images, the conventional CNN is not directly applicable to semi-regular geometric mesh surfaces, on which the cerebral cortex is often represented. In order to apply the CNN to surface-based brain research, we propose a geometric CNN (gCNN) that deals with data representation on a mesh surface and renders pattern recognition in a multi-shell mesh structure. To make it compatible with the conventional CNN toolbox, the gCNN includes data sampling over the surface, and a data reshaping method for the convolution and pooling layers. We evaluated the performance of the gCNN in sex classification using cortical thickness maps of both hemispheres from the Human Connectome Project (HCP). The classification accuracy of the gCNN was significantly higher than those of a support vector machine (SVM) and a 2D CNN for thickness maps generated by a map projection. The gCNN also demonstrated position invariance of local features, which rendered reuse of its pre-trained model for applications other than that for which the model was trained without significant distortion in the final outcome. The superior performance of the gCNN is attributable to CNN properties stemming from its brain-like architecture, and its surface-based representation of cortical information. The gCNN provides much-needed access to surface-based machine learning, which can be used in both scientific investigations and clinical applications.

## Introduction

In the machine learning domain, the convolutional neural network (CNN; LeCun et al., 1998; Krizhevsky et al., 2012) has made an enormous impact on pattern recognition. This approach utilizes replicated (shared) local filters in a convolution layer analogous to the tiled receptive fields in the hierarchical visual system of the brain, which make it efficient in detecting common local features regardless of their position in the image space. The CNN also takes advantage of hierarchical architecture by utilizing a pooling layer that represents distributed local features as global patterns. Owing to its strength in hierarchical feature detection, the CNN has been used in not only image-based pattern recognition but also identifying patterns in three dimensions: in volume (Kamnitsas et al., 2015; Maturana and Scherer, 2015; Nie et al., 2016), in time (e.g., dynamic images; Ji et al., 2013; Huang et al., 2015), and in different modalities (Kamnitsas et al., 2015; Nie et al., 2016). Despite many variations, the CNN is most commonly used for recognition of patterns in a two-dimensional (2D) image space. However, the conventional CNN technique cannot be directly applied to data about the three-dimensional (3D) geometric surface space, such as data about cortical thickness as indicated by the cortical surface.

In brain research, brain morphometry and functionality are often represented in cortical surface geometry (Van Essen and Drury, 1997; Van Essen et al., 1998; Dale et al., 1999; Fischl et al., 1999a; MacDonald et al., 2000). The most promising aspect of the surface-based approach is the ability to explore cortical thickness, which can typically be represented on the surface (Fischl and Dale, 2000; Kabani et al., 2001; Kuperberg et al., 2003; Narr et al., 2005). For example, Park et al. (2009) showed the specificity of the surface-based cortical thickness representation compared with volumetric representation. Metabolic activity can also be efficiently evaluated at the cortical surface (Park et al., 2006; Greve et al., 2014). Despite the many advantages of cortical surface representation of brain structure and function, no efficient method for applying a CNN over the cortical surface has been proposed. Recently, application of a CNN to non-Euclidean manifolds has been introduced in the computer vision fields to classify objects according to their geometric shapes (Boscaini et al., 2015; Masci et al., 2015; Bronstein et al., 2016). However, those methods require specific algorithms that cannot easily be used by conventional CNNs.

In this article, we propose a simple geometric CNN (gCNN) that expresses data representation on a geometric surface and recognizes cortical distribution patterns. Although the method can be expanded over any surface shape, we focus on the spherical surface because of its simplicity. The cortical surface has the same topology as a spherical surface; consequently, the cortical surface is often treated as a spherical surface—for example, in surface-based registration across brains (Fischl et al., 1999b). The gCNN performs convolution and pooling over the spherical surface to capture hierarchical features on the surface. In order to demonstrate the performance of the proposed method, we applied gCNN to sex classification using 733 cortical thickness maps from the Human Connectome Project (HCP; Van Essen et al., 2012). We compared the classification accuracy of the gCNN with those of a conventional support vector machine (SVM) and a conventional 2D CNN for thickness maps after projecting cortical thickness into the 2D image space (pulse-coupled neural network, pCNN).

Finally, we evaluated the performance of the gCNN in detecting position-invariant local features compared with the pCNN by testing the reusability of the low-level features after global rotation of thickness distribution at angles of 45° and 90°.

## Materials and Methods

### Geometric CNN (gCNN)

**Figure 1:**
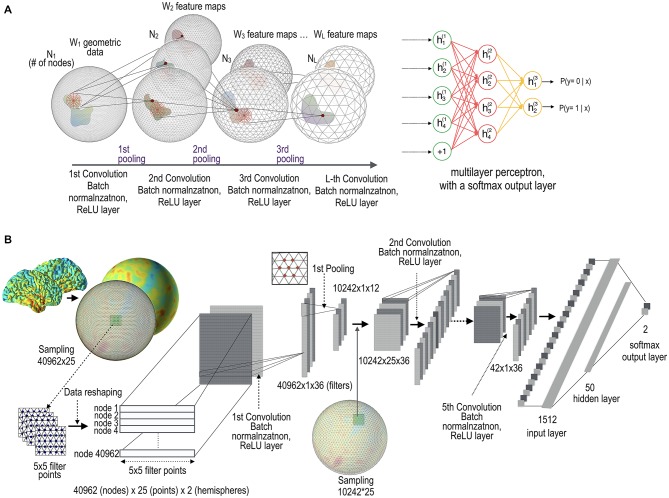
Architecture of geometric convolutional neural network (gCNN). **(A)** Conceptual architecture of the gCNN. When data on the cortical surface enter the convolution layer with batch normalization and rectified linear unit (ReLU) output function layers, W feature maps (corresponding to the number of filters at each convolution layer) are generated. The dimension of the nodes decreases from N1 to NL as the data pass through the pooling layers. The gCNN ends with a multilayer classifier. **(B)** Implementation level architecture of the gCNN. The input data 40,962 (nodes) × 25 (filter sample points) × 2 (hemispheres) are convolved with 36 filters, which are reduced to 42 (nodes) × 36 (filtered outputs, i.e., features) after five convolution and pooling steps. As the data pass through the layers, the number of features increases but the dimension of the nodes decreases. Finally, the convolution-pooling data enter the fully connected multilayer perceptron comprising a hidden layer with 50 nodes and a softmax output layer with two nodes.

The main units comprising the gCNN are surface-based convolution layers and pooling layers. The functions of these layers are similar to those of conventional CNNs (Krizhevsky et al., 2012; Chatfield et al., 2014; LeCun et al., 2015), with the exception that they deal with surface data. In order to utilize available conventional CNN toolboxes, we added data reshaping steps to each layer. Figure 1 illustrates the conceptual architecture and the implemented architecture of the gCNN. The architecture comprises an input data layer, mesh convolutional layers with data reshaping, batch normalization layers (Ioffe and Szegedy, 2015), rectified linear unit (ReLU) layers (Glorot et al., 2011), mesh pooling layers, and a fully connected layer with a softmax output function.

### The Convolution Layer

**Figure 2:**
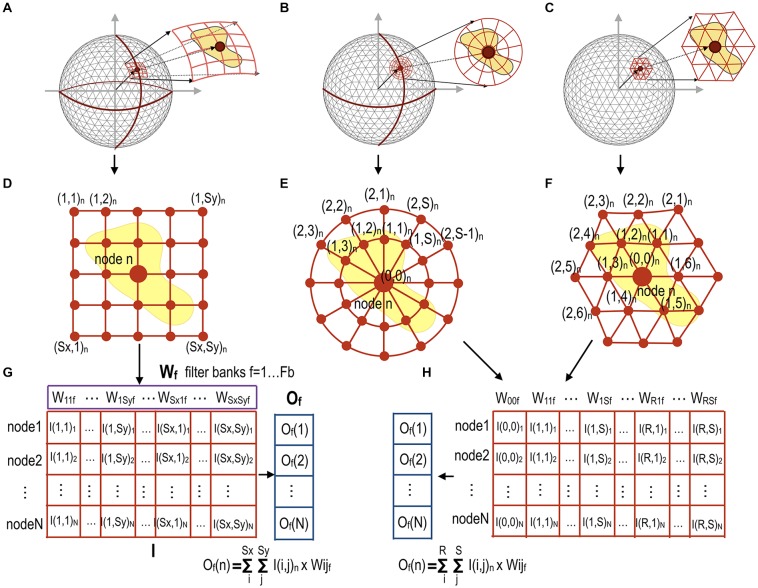
Data sampling and reshaping methods for three different types of filters according to patch geometry for mesh convolution. For all nodes in the sphere **(A)** rectangular filter points in a patch for each node *n* (Sx × Sy points for each node) **(D)** compose a row vector of a full-node filter point matrix **I** with dimensions [N × (Sx × Sy)] in **(G)**. A circular filter point matrix **(B)** can be similarly constructed by sampling circular points over the surface **(E)**. Polygonal filter points in **(C)** are composed of up to R-th order neighbor nodes **(F)**. A circular patch in **(E)** and a polygonal patch in **(F)** at each node compose a full-node filter point matrix I respectively, as shown in **(H)**. The convolution operation can be performed by multiplication of the filter point matrix **I** by a filter weight vector **W**_f_, resulting in output vector **O**_f_ for the filter weight vector f. Fb is the number of filters for each convolutional layer. These filtered data are down-sampled in the next pooling layer.

The convolution filter can be defined according to the patch geometry (i.e., the sampling methods over the mesh) of the filter. For each node in the mesh, the proposed method provides three types of filter according to spatial patch geometry: rectangular, circular, and polygonal patch grids (Figure 2). Each patch has spatially distributed sampling points (filter points) arranged at regular intervals around the node and subject to the convolution operation. A rectangular filter processes every patch composed of rectangular points. A circular patch has points centered on a mesh node and arranged radially. A polygonal filter convolutes inputs from the neighboring nodes for the target node.

Rectangular and/or circular patches for each node in the sphere were obtained by projection and rotation of rectangles and circles in the 2D space; e.g., the rectangular grids in the 2D space were first projected into the 3D spherical space, followed by spherical rotation to locate the origin of the rectangle at each spherical node by aligning the main axis of the rectangular grid along the geographical latitude. Circular patches were generated in the same way as rectangular patches. The patch size and distance between patch grid points can be chosen empirically by considering the coverage of the patch and computational and memory costs owing to the number of grid points.

To reduce computational loads, the intensity value corresponding to the filter points on the mesh surface is obtained by interpolating the intensity value of the nearest neighbor nodes to which the filter points belong.

In the conventional CNN, each image patch is convoluted with a filter in a sliding window manner. In order to utilize conventional CNN toolboxes using GPUs, we rearranged the sampled filter points to render the convolution operation as a simple filter weighting process (Figure 2). For each node n on the surface with a total node number N, filter points sampled from the rectangular, circular, or polygonal grid of the node are first reshaped into a row vector in the full-node filter point matrix **I** (dimension: number of nodes × number of filter points). The output vector **O**_f_ (dimension: number of nodes × 1) is obtained by multiplying the filter point matrix **I** by the *f*-th mesh filter vector **W**_f_ (dimension: number of filter points × 1), **O**_f_ = **I** × **W**_f_, *f* = 1, …, Fb (total number of filters at each convolution layer). Figure 2A shows an example of a rectangular patch for each node, which samples the surface data at rectangular filter points at regular intervals to create the **I**(i, j) matrix (Figure 2D). We multiply the sampled intensity **I**(i, j)_n_ (or thickness in the current study) obtained for each node by a filter weight vector **W**_f_, which generates an output [**O**_f_(*n*)] corresponding to node n (Figure 2G). The filter weight vector **W**_f_ (*f* = 1, …, Fb) is updated to optimize performance while training gCNN.

A circular patch composed of multiple circles for each node can also be constructed, as shown in Figures 2B,E. Patches at all nodes construct a full-node filter point matrix, as shown in Figure 2H. Figures 2C,F show a polygonal patch composed of the first-order and the second-order neighbor nodes (as filter points) of the corresponding node.

These different types of patch geometries can be used on a case-by-case basis. Circular and polygonal patches may be appropriate in some specific applications where the divergence of a node over the surface is important. In the current study, we used the rectangular filter point grid for pattern classification of cortical thickness, because our preliminary test showed the best performance with the rectangular filter in the sex classification. However, we can also choose circular and polygonal patches depending on the application.

### The Mesh Pooling Layer

**Figure 3:**
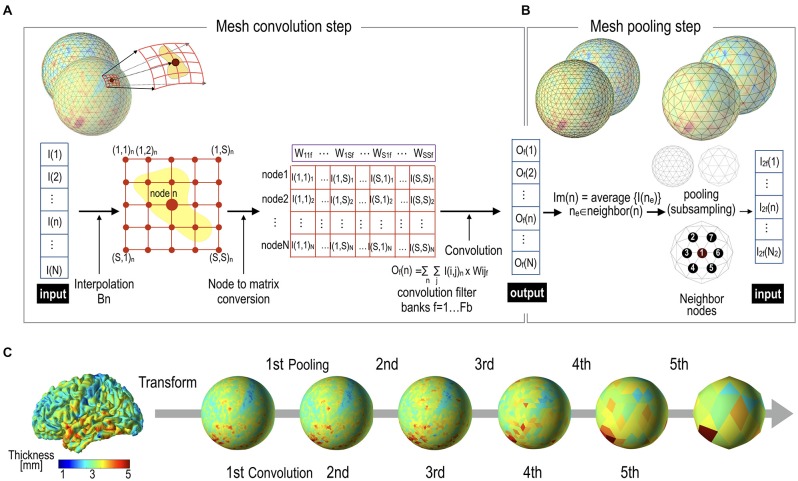
Mesh pooling after mesh convolution operation. **(A)** A mesh convolution layer processes inputs and generates outputs by multiplication of filter vectors with reshaped thickness data. **(B)** The output value of the convolutional layer is subsampled at the pooling layer. In this study, mean pooling was used. **(C)** Cortical thickness representation example of successive pooling processes. Cortical thickness data over a realistic cortical sheet (node size = 32,492) are transformed to a spherical surface. In order to utilize the regularity characteristic of the icosahedron, we interpolated 32,492 nodes into 40,962 nodes, which were subsequently subsampled to 10,242 (2nd), 2562 (3rd), 642 (4th), 162 (5th), and 42 (6th) spherical nodes, in the direction from local to global.

**Figure 4:**
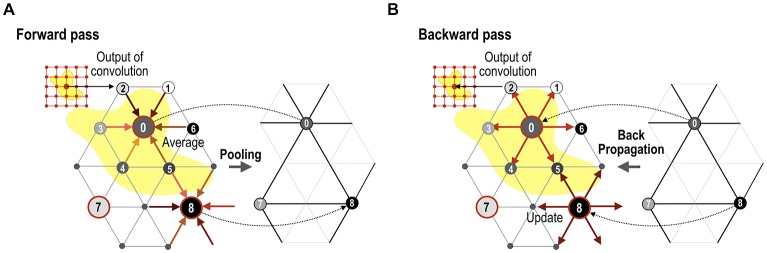
Forward mesh pooling and error backpropagation step. **(A)** Currently, the gCNN uses a mean pooling method that assigns the average value of the neighbor nodes to the target parent node. After pooling, only parent nodes remain (0, 7 and 8 in this illustration), and theses compose a less dense spherical surface. **(B)** When backpropagation is performed, the error is distributed equally to all the neighboring (child) nodes.

The convolution output is subsampled in the subsequent pooling layer (Figure 3). For pooling, we utilize the regularity characteristic of the icosahedron that can be expanded easily by a simple rule. The icosahedron increases the number of nodes by adding a new node to each center of three triangular edges, which divides one parent triangle into four child triangles. Iterating this process creates a fine spherical surface with node numbers 42, 162, 642, 2562, 10,242, 40,962 and so on. Subsampling can be performed in the reverse order of icosahedron expansion, leading to the spherical surface of the pre-expansion stage. Figure 3C shows an example of pooling over the icosahedron spherical system. In the current study, we used a mean pooling over the mesh structure during the forward propagation, which assigns the parent node with an average of the convolution outputs at the child nodes (Figure 4A). During the error backpropagation, errors in the parent nodes are evenly distributed to their child nodes, as illustrated in Figure 4B.

## Implementation of gCNN

To implement the proposed method, we modified the MatConvNet toolbox (Vedaldi and Lenc, 2015) available from http://www.vlfeat.org/matconvnet/. We constructed the gCNN by repeating the mesh convolution and pooling layers. Between the convolution and pooling layers, we inserted a batch normalization unit and a ReLU to increase the training performance. Semi-batch training with a batch size of 50 increases learning performance and expedites network learning, according to Ioffe and Szegedy (2015). The ReLU layer naturally leads to sparse nodal activity (Glorot et al., 2011). We used average pooling, which exhibited better performance than max pooling in preliminary evaluations conducted in the current study. Thus, the main operational complex comprises a convolution layer, a batch normalization layer, a ReLU layer, and a pooling layer. The final unit is a fully connected multilayer perceptron with a softmax output layer. Conventional backpropagation and a gradient descent algorithm are used to update the model weights, which are implemented in MatConvNet. To minimize the overfitting problem, we adjusted the learning rate from 0.02 to 0.001 during the training process.

## Application to Sex Classification

In order to evaluate the performance of gCNN, we applied gCNN to sex classification using 733 cortical thickness maps of healthy young adults (328 males and 405 females, mean age: 28.74 ± 3.70 years), who had both resting state fMRI and cortical thickness maps from the 900 HCP database^1^. Cortical thickness maps for those subjects were extracted from 3T T1-weighted MR imaging data using FreeSurfer^2^ (Dale et al., 1999; Fischl et al., 1999a), details of which were described in Glasser et al. (2013).

The cortical surface has the same topology as a spherical surface; consequently, the cortical surface is often treated as a spherical surface—for example, in surface-based registration across brains (Fischl et al., 1999b). The mapping from an individual cortical surface to a spherical surface is done by inflating the cortical surface, while minimizing metric distortion so that distances and areas are preserved (Fischl et al., 1999b).

In the HCP database, the cortical thickness is evaluated over 32,492 nodes in each hemispheric cortical surface. We interpolated the 32,492 nodal thicknesses into 40,962 nodal thicknesses using bilinear interpolation to make the pooling steps simple based on the icosahedron architecture. For each node, we normalized each individual’s thickness data by demeaning (i.e., subtracting the average thickness (across entire brain) of the individual from the individual’s thickness values at the node). The test data were also normalized by demeaning. In the first convolutional layer, we resampled 25 rectangular grid (i.e., a patch) points (5 × 5) from each node of the surface, which resulted in a full-node filter point matrix comprising 40,962 nodes × 25 points thickness data for each hemisphere (Figure 1). The patch size 5 × 5 was chosen empirically by considering the node resolution and spatial extent of the patch.

Both the left and right hemispheric cortical thickness maps were combined to create an additional dimension. We used 36 convolution filter banks with size (25 × 1) in the first layer. These filter banks were then convolved with a filter point matrix, with a size of 40,962 (nodes) × 25 (reshaped thickness filter points) × 2 (hemispheres), as shown in Figure 1.

## SVM of the Cortical Thickness

In order to compare the proposed method with conventional classifiers, we conducted SVM classification using LIBSVM (Chang and Lin, 2011). In order to optimize the SVM, we evaluated five types of SVM classifiers (C-SVC, nu-SVC, one-class SVM, epsilon-SVR and nu-SVR) with four different kernel types (linear, polynomial, radial basis function and sigmoid). The best-performing classifier and kernel were the C-SVC classifier and linear kernel with a kernel regulation parameter *C* = 1, epsilon: 0.001.

## Conventional CNN for Projected Cortical Thickness Images

Surface-based representation has often been projected into a 2D image, for example, as is done for maps of the earth. Similarly, pattern classification of cortical thickness can be conducted in the 2D image space after projection. In order to compare the gCNN with a conventional CNN of projected thickness images (hereafter, called pCNN), we projected the cortical thickness of the spherical surface onto the 2D image. Although there are many 2D projection methods for spherical data, we conducted projection by latitude and longitude. The portion of the non-cortical brain was set to zero in the training and testing processes. All cortical thickness data were projected into 224 × 224 images. In order to utilize continuous information (continuous over the boundary) in the spherical data, we used the marginal five pixels from the other side of the image for padding (the filter size of the first convolution layer was 11 × 11; thus, at least five pixels were needed for convolution). We adopted the CNN architecture from ImageNet-VGG-F (Chatfield et al., 2014), which has six convolutional layers (with a normalization layer and an activation layer (ReLU) for each convolution layer) and a fully connected softmax classifier layer.

## Performance Evaluation

In order to evaluate the performance of the classifiers (gCNN, SVM and pCNN), we divided 733 thickness samples into a training-validation set (670 samples) and a test set (63 samples). Using the training-validation set, we conducted 10-fold cross-validation, by splitting the thickness dataset randomly into 90% for training the model and 10% for validating the model. During the cross-validation, we optimized a model of each fold and evaluated the performance of the trained model using the test set. Based on the ratio of males and females (328 males and 405 females used in this study), the average numbers of males and females in the validation set of a fold (67 samples in each fold) were chosen as 29.6 and 37.3, respectively. The numbers of males and females in the test set were 28 and 35, in a male-to-female ratio similar to the entire data set.

We trained a model for each fold iteratively for a total epoch (or iteration) size of 40. When model training was not saturated (i.e., the difference in error rates between consecutive epochs is not 0) after 40 epochs, we extended training up to 70 epochs. All gCNN models (one model per fold) were saturated before 40 epochs in the experiment. Meanwhile, most pCNN models were saturated after 40–70 epochs.

**Figure 5:**
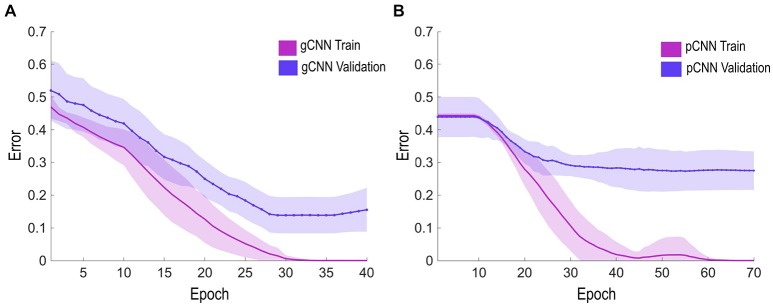
Error rate learning curves during the training and validation epochs. The solid line represents the average error rate of 10 models (for 10-fold validation), and the shaded region represents standard deviation of the models at each epoch. **(A,B)** show the learning curves of the gCNN and pulse-coupled neural network (pCNN). The gCNN was trained earlier and had superior performance over the pCNN.

We optimized the gCNN and pCNN models using a stochastic gradient method with a learning rate of 0.02. The error rate for each training epoch decreased as training proceeded (Figure 5). When the error rate curve was saturated, we changed the learning rate from 0.02 to 0.001 for fine tuning. To avoid the overfitting problem, we chose the epoch with the lowest error rate in the validation set, even before the error rate curve of the training set became saturated (called “early stopping”). We evaluated the accuracy of the optimized model for each fold in classifying the test data set.

For the statistical evaluation of the classification performance of the three classifiers (gCNN, pCNN and SVM), we conducted a one-way analysis of variance (ANOVA) of the classification accuracies at 10 folds with Bonferroni correction as a *post hoc* adjustment.

## Position Invariance of Local Features

**Figure 6:**
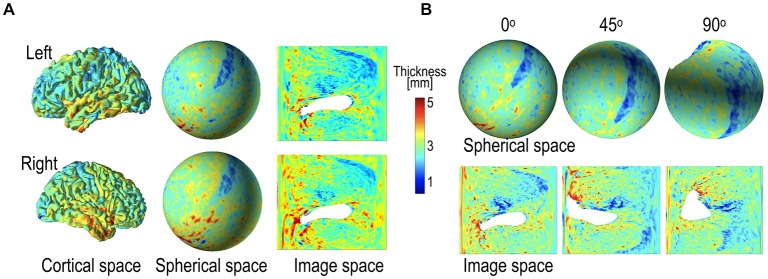
Example of cortical thickness representation over various geometry and thickness representation after global rotation (45° and 90°). **(A)** Cortical thickness defined in the cortical surface can be transformed into the spherical space and 2D image space. The thickness map in the 2D image was generated by projecting the spherical thickness map onto the image space based on latitude and longitude. **(B)** Spherical rotation of cortical thickness map changes representation on the sphere and images; the original, 45° rotation, and 90° rotated spheres and their projected images are presented. The global rotation considerably influences the projected images, while the local pattern in the spherical map is maintained after rotation. The white areas indicate where thickness measurement is not available.

As a successor of CNN, gCNN may have the position invariance property of detecting local features in the brain–a lower-level feature detector can often be used regardless of global position. To show this position invariance property, we conducted the same classification steps described above after globally rotating the spherical maps 45° and 90° (Figure 6). Instead of retraining all layers in gCNN, we reused weights from the trained model in up to 20 layers (five sets, each comprising a complex of convolution layer, batch layer, ReLU layer, and pooling layer, Nos. 1–20 of Table A1 in the Appendix) out of the 25 layers from the bottom. Only the four upper layers of the model (a batch normalization layer, a ReLU layer, a hidden layer, and an output layer) were retrained with two rotated datasets. In this evaluation, we did not conduct fine-tuning of the reused layers. Similarly, we reused model weights from up to 15 layers out of 19 layers in the pCNN (Nos. 1–15, Table A2 in the Appendix). The levels of gCNN and pCNN reused in this evaluation were chosen before the fully connected softmax-classifier set. By reusing lower layers, the training time was significantly reduced.

In 2D projections, there were severe distortions in the local features, particularly in the area of the poles (Figure 6B). The 2D projection also led to discontinuity in the boundaries (0° or 360° in longitude, −90° or 90° in latitude). Although we tried to rectify this weakness by padding regions across the circular boundary, this may not have been sufficient. We surmised that the local features might not be maintained after global rotation in the 2D projected image. We compared the performances of gCNN and pCNN after rotation. The details of the model structures for gCNN and pCNN are presented in Tables A1, A2 in the Appendix.

## Results

**Figure 7:**
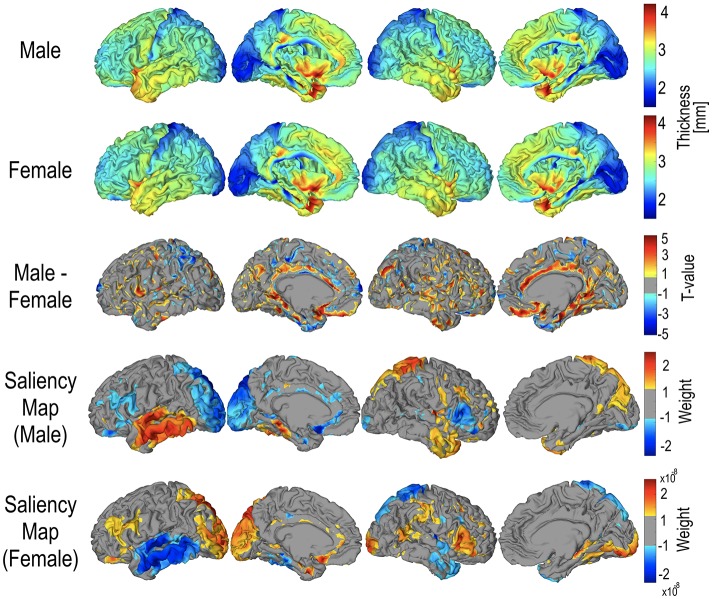
Mean cortical thickness maps for male (*N* = 328) and female (*N* = 405), and their statistical difference. For statistical differences, only areas with *p* < 0.05 are presented. Areas that were thicker in males are represented in red, and areas that were thicker in females are represented in blue. The mean saliency map was calculated by averaging the saliency weights of each individual in the group.

Figure 7 displays the group-average, group-differential (t-statistic) surface-based cortical thickness maps and salience maps used in the current study. In order to visualize feature importance (i.e., data representation) in the sex classification, a salience map for each individual was constructed from the trained gCNN according to Simonyan et al. (2013). The group average pattern of important feature distribution is slightly different from the pattern of group-level sex differences (Figure 7).

**Figure 8:**
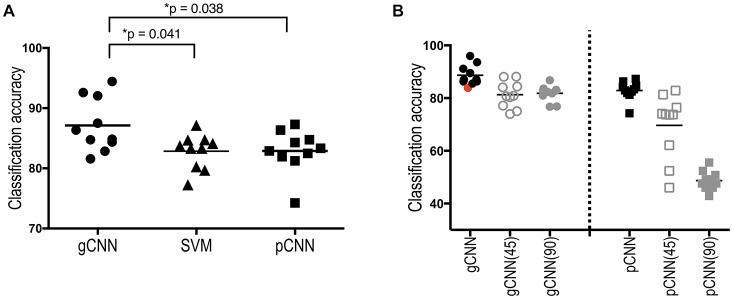
Sex classification accuracies of the gCNN, support vector machine (SVM), and pCNN, and classification accuracy changes after global rotation. **(A)** When classification accuracy was evaluated using 10-fold cross-validation, a significantly superior performance was found in the gCNN (mean = 87.14%, standarddeviation (STD) = 4.42) compared to the SVM (82.84%, STD = 2.91, *p* = 0.038) and the pCNN (82.54%, STD = 3.58, *p* = 0.041) according to one-way analysis of variance (ANOVA) with Bonferroni correction as a *post hoc* adjustment. **(B)** Classification accuracy did not significantly decrease after rotation in the gCNN (45°: 81.27%, STD = 4.98, 90°: 81.81%, STD = 3.06) but significantly decreased in the pCNN (45°: 69.64%, STD = 12.2, 90°: 48.73%, STD = 3.59) after rotation. The model with the red-point fold (the 7th fold) was used as the initial (reused) model (82.85%) of the gCNN for the position invariance test, as it exhibited similar accuracy to the pCNNs’ mean accuracy.

The classification performance of the gCNN, SVM and pCNN are summarized in Figure 8. The one-way ANOVA of the classification accuracy showed a significant main effect (group difference); *F*_(2,27)_ = 4.472 (*p* = 0.021). The average classification accuracy of the gCNN was 87.14% (standard deviation (STD) = 4.42), which is significantly higher than that of the SVM (mean = 82.84%, STD = 2.91, corrected *p* = 0.044) and the pCNN (82.54%, STD = 3.58, corrected *p* = 0.047). The pCNN and SVM showed no significant difference in their classification accuracy.

### Classification Performance of the Rotated Model

In order to evaluate the position invariance of the local feature detection, we chose a 7th-fold model (red point in Figure 8B) of the gCNN as an initial model because the accuracy of that fold gCNN model (82.86%) was close to the mean accuracy of pCNN (82.54%). Using outputs at the 20th layer in gCNN models (or the 15th layer in pCNN models) for the input thickness data, we conducted 10-fold cross-validation of the remaining upper layers with a softmax classifier. The average accuracies for the gCNN after rotation were 81.3% (STD = 4.98) and 81.8% (STD = 3.06) for 45° and 90° rotations, respectively, as shown in Figure 8B. This accuracy after rotation is not a significant decrease from the initial model accuracy of 82.86%. On the other hand, the pCNN showed average accuracies of 69.6% (STD = 12.20) and 48.7% (STD = 3.59) for the 45° and 90° rotations, respectively, which were significantly lower than the original accuracy for the 45° rotation (82.89%, STD = 3.58), and within a statistically similar level of probability for the 90° rotation.

## Discussion

In this article, we proposed a gCNN to evaluate neuroimaging data on the cortical surface. To show its usefulness in surface-based representation, we applied the gCNN to sex classification based on cortical thickness. The proposed method exhibited superior performance over the existing classification method (SVM) and conventional 2D CNN (pCNN) for cortical thickness mapping. It also exhibited minimal performance deterioration after global shifts, which implies that the local features in the gCNN are reusable.

### Surface-Based Methodology

The human cortex has a surface geometry, which renders the representation of neurometric features (e.g., cortical thickness) that are important for various neuroimaging researches. Surface representation is also advantageous in registering different brains for spatial normalization (Fischl et al., 1999b), registration between T1-weighted images and fMRIs or diffusion weighted images (Greve and Fischl, 2009), efficient spatial smoothing, and partial volume correction of functional or metabolic imaging data (Park et al., 2006; Greve et al., 2014, 2016). Because these surface-based processing steps are efficient in preprocessing, removing statistical confounding factors and thus enhancing statistical power, surface-based analysis has been widely applied in diverse brain studies exploring morphometry (Landin-Romero et al., 2017), thickness (Goldman et al., 2009; Park et al., 2009; Rimol et al., 2012; Van Essen et al., 2017), myelination (Glasser and Van Essen, 2011; Van Essen et al., 2017), metabolic activity (Park et al., 2006), and tau and amyloid PET scans (Cho et al., 2016). These advantages of surface-based representation necessitate the development of a surface-based method for machine learning applicable to cortical neuroimaging data.

### CNN and gCNN

The gCNN inherits the benefits of the CNN (LeCun et al., 1998; Krizhevsky et al., 2012). Local filters in the convolution layer of the CNN are characterized by sparse connectivity and shared weights across patches. A “replicated” local filter unit is effective in detecting common local features regardless of their position in the image space. Furthermore, sharing weights increases learning efficiency by reducing the number of parameters that need to be trained. CNN also takes advantage of a hierarchical architecture, which entails multiscale information processing from local regions to global regions. This hierarchical and multiscale architecture for information abstraction is implemented by the pooling layer in the CNN. As a type of CNN, gCNN is efficient in detecting features hierarchically, which may explain the superior performance of gCNN over SVM and pCNN.

### Sex Differences of Cortical Thickness

To demonstrate the performance of gCNN in this study, we presented an example of a sex classification problem based on cortical thickness. In previous cortical thickness analyses of sex, several brain regions showed cortical thickness differences between males and females. For example, Sowell et al. (2007) reported significant cortical thinning in males compared to females at the right inferior parietal lobe and right posterior temporal regions and a tendency of thinning at the left ventral frontal and left posterior temporal regions. Other studies have shown increased cortical thickness in females compared to males in the frontal lobe and the parietal lobe (Nopoulos et al., 2000; Allen et al., 2003). Studies measuring gray matter density and cortical thickness have also shown local increases in gray matter in women, primarily in the parietal lobes (Good et al., 2001; Narr et al., 2005) and both the parietal and temporal lobes (Im et al., 2006; Luders et al., 2006). All of these studies were based on group data and showed diverse brain regions having different cortical thicknesses according to sex. Thus, it is not clear whether this finding can be applied to sex identification of an individual. In the current study, using 733 sets of data, the gCNN utilized patterns of cortical thickness distribution to classify sex with reasonable accuracy. Furthermore, the pattern of saliency distribution differed from the group level *t*-test results for the thickness differences (Figure 7). The saliency maps were more similar to the results of Sowell et al. (2007). The advantage of the gCNN over the mass univariate *t*-test approach is its ability to detect multivariate patterns of cortical thickness and its robustness to noise effects. This study confirms that the gCNN can find features that summarize differences between groups.

The sex classification using cortical thickness may not be of a practical use that shows the clinical utility of this new machine learning algorithm. Nevertheless, the classification of sex based on the cortical thickness may be a good test-bed for different classifiers with a balanced data set (male and female). Indeed, the sex classification is not a trivial problem as reflected in the relatively low accuracy of conventional classification methods (e.g., less than 85% in SVM). Furthermore, the sex classification with a large-sized (HCP) database and a balanced number of class samples (e.g., man and woman) provides us a chance to train a gCNN model for the purpose of potential reuse beyond sex-classification. In most classification studies with cortical thickness data, we may not have a sufficiently large number of data to train deep layers in the gCNN. A gCNN model for sex-classification, if trained well using a large dataset, may be reused in diverse applications, which is discussed in following section.

### Position Invariance in gCNN

In the hierarchical architecture of gCNN (as well as CNN), the lower-level filters are considered to detect features that are common to diverse applications, whereas global features at higher levels are more specific to each application. Therefore, lower-level feature detectors, which require sufficient data to train, can be reused for applications other than that for which the model was trained without significant distortion in the final outcome. After reusing the lower-level feature detectors, only the upper layer may be trained for the data with new application. This could not only reduce the computational cost but also mitigate the problem of insufficient data. We partially demonstrated this problem by shifting the global positions of cortical thickness while maintaining local properties. Compared to a conventional CNN with a 2D projected thickness image, the gCNN shows a consistent level of accuracy after global rotations of thickness maps. This position invariance test suggests that we can reuse the lower-level feature detection of the gCNN (found in the sex classification, for example) for the new applications, thus mitigating the need for a large number of samples for training from the beginning.

Instead of a gCNN, one may consider a 2D projection of cortical thickness for conventional CNN applications, as has been done in EEG analysis (Bashivan et al., 2015). As shown in the rotation example, a 2D projection of 3D surface representation leads to inevitable distortion in representing common local features according to location. In particular, shapes near the pole are largely different from shapes at the equator. This violates the position invariance of the CNN in describing a common set of local features. The distortion during 2D projection might be reduced by utilizing surface-based flattening (Fischl et al., 1999a); however, there are still problems associated with cutting the surface into the 2D image space. Flattening inevitably causes splits in the continuous representation, relocating close areas to distant areas, which may hinder model-reuse for different applications. Instead of requiring an additional flattening step, the gCNN can be directly applied to the surface-based data using conventional CNN toolkits with slight modification.

### gCNN for Surface-Based Representation

The classification performance depends on how well the model is optimized. There are many factors that can be optimized in both a gCNN and a pCNN, such as model structure, training strategy, and data augmentation, which should be chosen empirically depending on the application. The current study presented a gCNN example that exhibited superior performance compared to an SVM and a pCNN in sex classification, possibly owing to advantages inherent in the CNN, taking advantage of hierarchical feature detection and utilizing geometric information in the model without significant distortion.

Nevertheless, the purpose of the current study is not to show the general superiority of the gCNN over the other methods, as the performance may vary according to the quality of optimization. Instead, the current study is aimed at introducing a novel and simple CNN scheme that can easily be applied to surface-based or mesh-based neuroimaging data with reliable accuracy.

The gCNN differs from previous variants of 3D CNN in dealing with surface-based point data. In the classification of geometric shapes, previous studies transformed 3D points into voxels in the volume space, to which 3D CNN was applied. Examples include 3D ShapeNets (Wu et al., 2015), VoxNet (using an occupancy grid; Maturana and Scherer, 2015), PointNet (Garcia-Garcia et al., 2016), and LightNet (Zhi et al., 2017). In contrast to voxel-type 3D applications, the gCNN processes values (thickness in the current study) of the 3D points in the surface geometry while maintaining its geometry.

The gCNN shares the basic mathematical framework of geodesic CNN formulated for geodesic shape classification on non-Euclidean manifolds (Boscaini et al., 2015; Masci et al., 2015; Bronstein et al., 2016). Although the gCNN captures the pattern of feature (thickness) distribution in the same non-Euclidean space, if not the pattern of geometric shape features, gCNN can be considered a specific type of non-Euclidean CNN. The advantage of the gCNN over previous non-Euclidian CNNs (requiring complex geometric processing steps) is its technical simplicity achieved by a data sampling and reshaping method over a sphere, which is easily applicable to conventional software toolboxes implemented on GPUs without significant modification of the source code.

In addition to application with cortical thickness data, the gCNN can be extended to various applications with diverse neuroimaging data such as fMRI or PET images after surface mapping. The gCNN can also be used with multi-layered input, concatenated with different modalities or different cortical (three or six) layer information over the same cortical surface architecture.

In conclusion, the gCNN takes advantage of both the CNN properties stemming from its brain-like architecture and a surface-based representation of cortical information. The gCNN may expedite surface-based machine learning in both scientific and clinical applications.

## Ethics Statement

Data were provided in part by the Human Connectome Project, WU-Minn Consortium (Principal Investigators: David Van Essen and Kamil Ugurbil; 1U54MH091657) funded by the 16 NIH Institutes and Centers that support the NIH Blueprint for Neuroscience Research; and by the McDonnell Center for Systems Neuroscience at Washington University.

## Author Contributions

H-JP initiated the current study and developed the main idea and initial code. S-BS implemented codes in detail and conducted evaluations of the proposed method. CP prepared the database and preprocessed the data.

## Conflict of Interest Statement

The authors declare that the research was conducted in the absence of any commercial or financial relationships that could be construed as a potential conflict of interest.

## Footnotes

^1^https://www.humanconnectome.org/

^2^https://surfer.nmr.mgh.harvard.edu

## Appendix

**Table A1:** gCNN architecture and parameter size.

No.	Type	Input size	Patch size/stride	Output size	Param. mem.
1	Convolution 1	40962 × 25 × 2	1 × 25/1	40962 × 1 × 36	7 KB
2	Batch normalization 1			40962 × 1 × 36	576 B
3	ReLU 1			40962 × 1 × 36	
4	Mean pool 1	40962 × 1 × 36	1 × 6 or* 1 × 7/1	10242 × 1 × 36	
5	Convolution 2	10242 × 25 × 36	1 × 25/1	10242 × 1 × 36	127 KB
6	Batch normalization 2			10242 × 1 × 36	576 B
7	ReLU 2			10242 × 1 × 36	
8	Mean pool 2	10242 × 1 × 36	1 × 6 or 1 × 7/1	2562 × 1 × 36	
9	Convolution 3	2562 × 25 × 36	1 × 25/1	2562 × 1 × 36	127 KB
10	Batch normalization 3			2562 × 1 × 36	576 B
11	ReLU 3			2562 × 1 × 36	
12	Mean pool 3	2562 × 1 × 36	1 × 6 or 1 × 7/1	642 × 1 × 36	
13	Convolution 4	642 × 25 × 36	1 × 25/1	642 × 1 × 36	127 KB
14	Batch normalization 4			642 × 1 × 36	576 B
15	ReLU 4			642 × 1 × 36	
16	Mean pool 4	642 × 1 × 36	1 × 6 or 1 × 7/1	162 × 1 × 36	
17	Convolution 5	162 × 25 × 36	1 × 25/1	162 × 1 × 36	127 KB
18	Batch normalization 5			162 × 1 × 36	576 B
19	ReLU 5			162 × 1 × 36	
20	Mean pool 5	162 × 1 × 36	1 × 6 or 1 × 7/1	42 × 1 × 36	
21	Fully connected layer 6	42 × 1 × 36	42 × 1/1	1 × 1 × 50	295 KB
22	Batch normalization 6			1 × 1 × 50	800 B
23	ReLU 6			1 × 1 × 50	
24	Fully connected layer 7	1 × 1 × 50	1 × 1/1	1 × 1 × 2	408 B
25	Softmax classifier	1 × 1 × 2			
Total					1.63 MB

**The size of the mean pool at each node differs according to the number of neighborhood nodes at the node, either 6 or 7*.

**Table A2:** pCNN architecture and parameter size.

No	Type	Input size	Patch size/stride	Padding	Output size	Param. mem.
1	Convolution 1	224 × 224 × 2	11 × 11/4	1	54 × 54 × 64	61 KB
2	ReLU 1	54 × 54 × 64			54 × 54 × 64	
3	Normalization 1	54 × 54 × 64			54 × 54 × 64	
4	Mean pool 1	54 × 54 × 64	2 × 2/2	0	27 × 27 × 64	
5	Convolution 2	27 × 27 × 64	5 × 5/1	2	27 × 27 × 64	400 KB
6	ReLU 2	27 × 27 × 64			27 × 27 × 64	
7	Normalization 2	27 × 27 × 64			54 × 54 × 64	
8	Mean pool 2	27 × 27 × 64	2 × 2/2	0	13 × 13 × 64	
9	Convolution 3	13 × 13 × 64	3 × 3/1	1	13 × 13 × 64	144 KB
10	ReLU 3	13 × 13 × 64			13 × 13 × 64	
11	Convolution 4	13 × 13 × 64	3 × 3/1	1	13 × 13 × 64	144 KB
12	ReLU 4	13 × 13 × 64			13 × 13 × 64	
13	Convolution 5	13 × 13 × 64	3 × 3/1	1	13 × 13 × 64	144 KB
14	ReLU 5	13 × 13 × 64			162 × 1 × 36	
15	Mean pool	13 × 13 × 64	2 × 2/2		6 × 6 × 64	
16	Fully connected layer 6	6 × 6 × 64	6 × 6/1	0	1 × 1 × 100	900 KB
17	ReLU 6	1 × 1 × 100			1 × 1 × 100	
18	Fully connected layer 7	1 × 1 × 100	1 × 1/1		1 × 1 × 2	408 B
19	Softmax classifier	1 × 1 × 2				
Total						1.79 MB
